# An unexpected alliance between stress responses to drive oncogenesis

**DOI:** 10.1186/s13058-014-0471-1

**Published:** 2014-11-06

**Authors:** Melissa M Keenan, Chien-Kuang Cornelia Ding, Jen-Tsan Chi

**Affiliations:** Department of Molecular Genetics and Microbiology, Duke University Medical Center, 268 CARL Building, Research Drive, Box 3054 DUMC, Durham, NC 27710 USA; Center for Genomic and Computational Biology, Duke Medical Center, 2177A CIEMAS Building, 101 Research Drive, DUMC 3382, Durham, NC 27708 USA

## Abstract

*XBP1* is a well-characterized regulator of the unfolding protein response that is activated in response to unfolded or misfolded proteins or nutrient deprivation. The conventional wisdom is that *XBP1* is activated to coordinate the unfolded protein response and promote cellular survival under stresses. A recent study provides intriguing evidence that, in triple-negative breast cancer, *XBP1* plays a major role in promoting oncogenesis and cancer stem cell properties. Unexpectedly, *XBP1* accomplishes this by recruiting hypoxia-inducible factor 1α and activating oncogenic transcriptional programs. This study reveals a surprising hierarchy and alliance between two stress regulators with distinct transcriptional outputs to promote an aggressive oncogenic state.

## Background

Cells within a solid tumor are constantly exposed to fluctuating physical and chemical conditions in the microenvironment, including pH dysregulation, oxidative stress, and nutrient deprivation [1]. To cope with these fluctuations, tumor cells demonstrate a wide spectrum of mechanisms that sense and respond to these stresses. For example, a low partial pressure of oxygen (pO_2_) level stabilizes the hypoxia-inducible factors (HIFs) and triggers a hypoxia response [2]. Similarly, various oxidative stresses promote the nuclear translocation of NRF2 to induce a set of genes that enhances oxidative-stress tolerance. Although these responses facilitate stress adaptations, many of these proteins and pathways also play an active role in promoting or repressing oncogenesis. For example, the HIFs [3] and NRF2 [4] can be oncogenic and their constitutive activation directly contributes to tumor development. Hypoxia pathway is more active in triple-negative breast cancers (TNBCs) than in other breast cancers [5]. However, the physiologic cause for enhanced HIF-1α protein levels leading to the elevated hypoxia response remains unknown since these tumors, as a group, do not have lower pO_2_ [5].

The IRE1-XBP1 pathway is one of the three branches of the unfolding protein response (UPR) that senses and responds to the accumulation of misfolded proteins in the endoplasmic reticulum (ER) caused by nutrient deprivation and other stresses. The transmembrane ER protein IRE1 senses these stresses and excises a 26-bp segment from the *XBP1* mRNA, converting the inactive unspliced *XBP1* to the active spliced *XBP1* (XBP1s) whose translated protein triggers the transcription of many UPR genes [6]. Since the IRE1-XBP1 pathway is considered an adaptive survival mechanism under stress, the inhibition of the UPR reduces cellular survival and tumor growth [7]. Therefore, therapeutic targeting of IRE1-XBP1 may hamper the UPR required for cancer cell survival under stress.

## The article

In a recent letter to *Nature*, Chen and colleagues [8] provided intriguing data to show that *XBP1* acts as a tumor driver that is required for oncogenesis and cancer stem cell phenotypes associated with TNBC. Unexpectedly, *XBP1* mediates its oncogenic properties by physically interacting with and recruiting HIF-1α to initiate the hypoxia response. The recruitment is essential to induce oncogenic and self-renewal phenotypes in TNBC. An *XBP1* gene expression signature identified by using ChiP-seq analysis significantly overlaps with the HIF-1α signature and is associated with poor prognosis in TNBC. Most importantly, epistasis analysis indicates that *XBP1* lies upstream of HIF-1α, occupying the regulatory regions and recruiting HIF-1α, via direct physical interaction, to the promoter regions of their shared target genes. Therefore, *XBP1* is required for a robust hypoxia expression program in TNBC. The authors conclude from these results that that *XBP1*s activation and promoter occupancy is not just a passive adaptive response for survival under stress, but rather is a driving oncogenic event in TNBC.

## The viewpoint

This article provides a potential explanation for the elevated hypoxia pathway activation in TNBC and breast cancer stem cells. Moreover, although both HIF-1α and *XBP1* are stress-responsive proteins, high baseline activities of both transcriptional factors can be found in TNBC without apparent stress exposure. What triggers the IRE1-XBP1 pathway in TNBC and breast cancer stem cells? One obvious candidate is hypoxia since it activates both *XBP1* and HIF-1α. However, the pO_2_ concentration needed to activate the UPR is much lower (pO_2_ < 0.01%) than what is needed to activate HIF-1α [9]. Furthermore, whereas hypoxia can be readily triggered by low pO_2_, *XBP1* is activated only by combined low pO_2_ and lactic acidosis [10]. Therefore, *XBP1* may be activated by combined metabolic stresses associated with the acquisition of cancer stem cell and TNBC properties. The higher UPR in TNBC is shown by a dilated ER [8] and increased sensitivity to hsp90 inhibitors that kill cells by hindering the UPR [11]. Such a high level of UPR in TNBC may be caused by increased protein production, higher oxidative stress, or lower levels of nutrients driven by vigorous glycolysis and altered glutamine metabolism [12]. Currently, TNBC is treated primarily by cytotoxic chemotherapies. Targeting the IRE1-XBP1 pathway, such as with an inhibitor of IRE1 (for example, STF-083010) [13], may have significant therapeutic value for TNBC.

Of course, these results also raise questions for further investigation. For example, why does the co-occupancy of *XBP1* and HIF-1α occur in TNBC but not luminal breast cancer cells? Such differences may be explained by TNBC-specific chromatin accessibility status or other available co-activator proteins. Specifically in TNBC, *XBP1* may serve as a ‘pioneer factor’ [14] that recruits other co-activators to trigger and maintain HIF-1α stability. A recent study has identified at least seven different subtypes of TNBCs with varying sensitivity to hsp90 inhibitors [15]. Therefore, it will be important to determine the extent of this XBP1-HIF-1α co-regulation among the subsets of TNBCs as well as cancer stem cells from other tumor types.

In conclusion, this study shows an unexpected dominant role for *XBP1* in the recruitment and activation of HIF-1α-driven oncogenesis in TNBCs. Like many other stress response pathways, these proteins are not just passive players to keep tumors alive under stress. Instead, stress response proteins can be oncogenic drivers that coordinate stress tolerance with other transcriptional factors to enhance and modulate their expression programs. Therefore, proteins such as HIF-1α and IRE1-XBP1 may be excellent targets in our efforts to treat cancers that have yet to benefit from other targeted therapeutics.

## Abbreviations

ER: Endoplasmic reticulum
HIF: Hypoxia-inducible factor
pO_2_: partial pressure of oxygen
TNBC: Triple-negative breast cancer
UPR: Unfolding protein response
XBP1s: spliced *XBP1*
